# Book Reviews

**Published:** 1828-08

**Authors:** 


### CRITICAL ANALYSES.

Quæ laudanda forent, et quæ culpanda, vicissim

Illa, prius, cretâ; mox hæc, carbone, *notamus*.—Persius.

*Transactions of the Medico-Chirurgical Society of Edinburgh.* Vol. III. Part I. With Plates.—8vo. pp. 316. Black, Edinburgh; Longman and Co. London. 1828.

So many interesting communications have been contained in the previous volumes of the Transactions of the Medico-Chirurgical Society of Edinburgh, that we naturally look in each succeeding part for additional proofs of professional zeal and ability. We hasten, therefore, to give an abstract of the present volume, which has just issued from the press.

The first paper is from Dr. Boggie, Surgeon to the Forces, upon the subject of *Hospital Gangrene, with reference to the disease chiefly as it appeared in the British Army during the late war in the Peninsula.*

Hospital gangrene may be regarded as the most serious affection to which wounded surfaces are liable. It destroys without distinction, and involves in one common mass all the textures which it attacks. In military hospitals this affection is seen in its most aggravated forms. No disease, indeed, is more destructive than this to an army on service. During the late war in the Peninsula, it was extremely fatal in proportion to the numbers affected by it, and many men were totally unfitted for the service by its ravages. Much diversity of opinion exists as to the nature of the affection, its causes, and treatment. Dr. Boggie, therefore, is induced to give an account of the disease as it occurred to himself, with a description of the treatment, and the probable causes which produce the malady. In the works of the oldest writers, we find allusion made to a gangrenous affection supervening to wounds and ulcers. The only doubt whether the disease mentioned by them is the same as hospital gangrene, seems to arise from their not having noticed its contagious nature. Dr. Boggie conceives that this is no proof of its not being the same disease, as the ancients were but little acquainted with the subject of contagion and infection, and that point even now is by some disputed.

"Hospital gangrene has been found to prevail very often in ships of war, and in naval hospitals, where great numbers of wounded have been crowded together. It has also existed in hospitals by no means crowded, and where every attention was paid to ventilation; and it has been known to prevail, and that extensively, among wounded who had never been in hospital, as I shall afterwards relate. It shows itself at all seasons of the year; but authors are not agreed as to that in which it chiefly prevails. Boyer seems to think that it is most frequent after the great heats of summer, and during a continuance of southerly winds.* I hope to be able to prove that it will be found in its greatest virulence during a continuance of very hot weather.

"There are two forms under which hospital gangrene usually appears. The first I would name Contagious Gangrene; the second Phagedæna Gangrænosa. Professor Delpech, of Montpellier, who has given a very excellent account of this disease, mentions four different forms: the first he denominates the Ulcerous (Ulcereuse); the second the Pulpous (Pulpeuse); the third and fourth seem to be varieties of these two.†

"When a wound or ulcer is affected with contagious gangrene, it becomes painful and swollen, loses its healthy florid appearance, and the granulations, which were small and distinct, become flabby, and appear sometimes as if they were distended with air; at other times, vesicles containing a watery coloured fluid, or bloody serum, have been observed, and the sensation in the sore has been described as resembling the stinging of a gnat. The secretion of pus is suspended; the wound is dry; and covered with a tenacious viscid ash-coloured matter, which adheres firmly to the surface. When this morbid state has existed for some time, a discharge take place of a thin ichorous matter, of a very peculiar smell; the pain increases, the edges of the wound are reverted, and in general assume a circular form; an erysipelatous redness surrounds the wound, and sometimes extends to a great distance, even over a whole limb; the neighbouring glands, as those of the axilla or groin, swell, inflame, and sometimes suppurate; febrile symptoms become apparent; the pulse is accelerated, full and strong; the heat of the surface is much increased; the patient complains of nausea and thirst; the tongue is covered with a whitish or brown crust; and the bowels are in general constipated. The inflammation goes on increasing, the thin ichor continues to be discharged in great quantity, and a thick slough, apparently of coagulable lymph, covers the whole surface of the wound; the fetor becomes intolerable, and the pain quite insupportable. In the last stage, there is in general an oozing of blood from the surface of the wound, and not unfrequently distinct hemorrhage, from the corrosion or destruction of the larger blood-vessels. Sphacelus takes place to a greater or less extent; the strength of the patient fails; the pulse sinks; his countenance becomes collapsed and altered; the skin is bedewed with a clammy sweat; and a diarrhœa with hiccup coming on, the scene very soon terminates.

* Vide Traité de Maladies Chirurgicales, tom. i. p. 321.

† Vide Memoire sur la Pourriture d'Hopital, p. 4, et seq.

"Though this is the most common form of the disease which I have seen, as it occurs in a recent wound, and in strong healthy men, who are the ordinary subjects of it, yet I am well persuaded that the fever which accompanies hospital gangrene is not always of so phlogistic a character. It has been often observed to partake more of a typhoid type; and it is of the utmost consequence in practice to attend to this distinction, as it will be found that what would be a valuable remedy in the one case might, if carried to any extent, be very pernicious in the other. The not attending sufficiently to this circumstance,—that is, to the phlogistic or typhoid type of the fever,—has, I am convinced, often led to fatal mistakes, and seems to be partly at least the cause of that great diversity of opinion among medical men regarding the best mode of treating this very dangerous affection." (P. 3.)

The other form in which hospital gangrene usually manifests itself is more of a chronic nature, appearing seldom in a recent wound. Such patients had generally been long in hospital, and many had suffered attacks of the more acute form, and when the wound was apparently doing well, the granulations healthy, secreting good pus, and sometimes even nearly cicatrised, a small dark-coloured spot, or ulceration, commonly appeared on the edge of the sore.

"This little ulceration was in general of a circular form, its edges ragged, its bottom unequal and excavated, and secreting a matter of a very peculiar smell. Ulcerations of the same kind not unfrequently appeared in other points, which, spreading in all directions, united, and soon extended over a great part of the wounded surface. At times this ulceration has been known to go on, and to cause very considerable destruction of parts, without the system appearing to be much affected by it; but most frequently, after it had spread to a certain extent, symptoms denoting constitutional irritation became apparent: these were nausea and loss of appetite, thirst, foul tongue, restlessness, a small and quick pulse, and heat of skin. After the febrile symptoms had appeared, the progress of the ulceration was more rapid, and very often extended beyond the limits of the original sore; the discharge became bloody, and the fetor peculiar to this affection more offensive. Sphacelus in many instances took place, and some time before death the same train of symptoms occurred already described as taking place in the last stage of the more acute form of the disease. This is the depascent, or phagedenic, form of hospital gangrene, or what may be called phagedæna gangrænosa." (P. 7.)

In the milder cases of this disease, the skin and cellular membrane are originally and principally concerned, and sometimes it may be confined to these textures. In more violent cases, one structure is destroyed after another. The bones even, being deprived of their covering, often become carious. The duration of the disease is various. It sometimes terminates as early as the third, fourth, or fifth day, either in recovery or death. Patients who have been once attacked, even when convalescent, are very liable to suffer a relapse. Dr. HENNEN mentions a case in which the patient survived twelve different attacks, but sunk under the thirteenth. Specific sores, such as venereal, scrofulous, and variolous, are thought to be less liable to this affection than simple sores. They are, however, by no means altogether exempt. Dr. Hennen mentions a case in point. The patient had an open bubo, and was carried off by the disease in forty-eight hours; the gangrene affecting the sore almost instantly, eroding the great vessels, and destroying the abdominal parietes to a great extent.

"A great peculiarity in the phagedenic form of the disease is, that different actions, such as the ulcerating, suppurating, and cicatrising, may frequently be seen going on in one sore at the same time. During the prevalence of the hospital gangrene at Bilboa, this peculiarity was often observed; the same thing was remarked by Dr. Rollo at Woolwich.

"After military punishments, in consequence of neglect or other causes, hospital gangrene sometimes occurs; and, from what has been stated in the description of the disease, the appearance and consequences of it may be easily imagined." (P. 9.)

In warm climates, phagedæna is very liable to ensue after punishment. Dr. Boggie states that a spirituous embrocation is the best application to heal the wounds thus produced, and to prevent the subsequent bad effects.

Hospital gangrene is considered to be nearly allied to erysipelas, if it be not a modification of that disease.

The causes which induce this affection are not yet determined. The foul air of crowded hospitals is doubtless a frequent cause. But we are told there is no hospital, however small, airy, or well regulated, where it may not prevail.* It has been known also to appear among wounded who had not been in hospital at all.† Particular states of the atmosphere have certainly some influence in the production of this formidable ailment. It has been known to prevail at all seasons of the year; but, according to Dr. Boggie's experience, more frequently and severely in hot weather than in cold. He is inclined to consider a heated atmosphere to be one of the most powerful exciting causes. Inattention to cleanliness

* Bell's Principles of Surgery, vol. i. p. 108.

† Rollo on Diabetes, vol. ii. p. 262.

produces the worst effects upon wounds and sores, and frequently produces gangrene, particularly the phagedenic form. Acrid or irritating applications contribute very effectually to the production of the disease. This is one objection to the use of ointments. When long kept, they always become rancid, and irritate extremely. On this subject, the suggestions of Sir EVERARD HOME are worthy of attention.*

*Stimulating food*.—Dr. Boggie has very little doubt that the disease may be produced by change of food, as from vegetable to animal diet. The abuse of wine and spirituous liquors is very likely to produce hospital gangrene.

"In that very violent form of hospital gangrene which prevailed at Bilboa in the summer and autumn of 1813, whatever other causes might have contributed to the production or continuance of the disease, there is not a doubt in my mind but that it was rendered much more virulent, and that it was even perpetuated in the hospitals there, by the use of wine and other stimulants, injudiciously administered. From an idea which very generally prevailed that the accompanying fever was typhus, and that hospital gangrene could not be prevented, or successfully treated, unless by stimulants, antiseptics, and tonics, a liberal allowance of wine was made to every patient as a preventive; and, when the disease actually appeared, it was then prescribed in increased quantity as a cure; the consequence of which was a fatal termination in almost every case. On this point I am very sorry to be obliged to differ so widely in opinion from my friend Dr. Hennen." (P. 19.)

Motion, or mechanical irritation, is a much more frequent cause of hospital gangrene than has been imagined. Dr. B. is convinced that, in transporting the wounded from the field, or from one hospital station to another, when at any considerable distance, more cases of hospital gangrene appeared upon the road than in any other situation.

*Specific contagion*.—When the disease is once produced, although the same causes continuing to operate may be sufficient to keep it alive, it appears probable that a contagion is generated, and that the disease may be propagated in this way to a certain extent at least, even although the causes by which it was originally produced should have ceased to act. Dr. Boggie, however, imputes much less to this than to the continued operation of the original or other irritating causes, although he feels assured that the disease is contagious, and even infectious. The prognosis, as in other diseases, must depend on circumstances.

"If the patient is young and healthy, of temperate habits, and the accompanying fever inflammatory, even although the affection

* Home on Ulcers, p. 39.

should be pretty severe, the prognosis, I think, may be favorable ; but, on the contrary, if the patient is old, addicted to intemperance, his health bad, and particularly if the gangrene should be complicated with fractures of the bones, or a scorbutic diathesis be present, with a fever evidently typhoid, it must be very unfavorable.

"In laying down rules for the treatment of this complaint, we must be guided entirely by the symptoms. If the constitution is much affected, general remedies will be required; if, on the contrary, the affection appears to be entirely local, the cure may be trusted to topical applications; but in many cases both the one and the other will be found to be necessary. It is of the utmost consequence, also, to attend to the type of the accompanying fever, and to ascertain whether the gangrene is simple or complicated with any other disease, such as scurvy or bilious fever, in which case its character will be modified, and the corresponding treatment must also be different." (P. 32.)

The treatment may be divided into general and local. When the wound is recent, and the patient young and healthy, the accompanying fever is mostly inflammatory. If the spirits of the men are dejected by great sufferings and frequent defeat, the fever is of a low type. In the former cases bloodletting will be required : it must, of course, be proportioned to the degree and violence of the inflammation, and the age and strength of the patient. In men not of a robust constitution, and who have lingered long in hospital, or suffered much from ill health, we must act with the greatest caution. Bloodletting in such is either altogether inadmissible, or should be sparingly used. To local bleedings, by cupping and scarifying, Dr. Boggie has no objection. "The danger of the punctures becoming gangrenous appears to me not to be very great. The same objection has been made to general bloodletting; but, though I have bled many in this disease, I never saw a single instance of gangrene supervening to the operation." (P. 34.)

As far as the observation of the author goes, in the early or inflammatory stage of hospital gangrene, spontaneous hemorrhage seldom occurs, and, if it be not too profuse, it might be beneficial. In the latter stages, he considers it an alarming symptom. Many patients at Bilboa were thus carried off. Upon this subject, Dr. B. differs from Dr. Hennen.*

Emetics have been sometimes used with advantage ; but as a general remedy they are considered inferior to cathartics. The cases to which they are chiefly applicable are those where

* Military Surgery, p. 223.

the stomach is loaded, and where the fever appears to be of a bilious character. By universal consent, cathartics are deemed indispensable. Their use is indicated where bleeding would have been improper, or at least not much required. Bark, in the *advanced* stages, may be given alone, or with mineral acids. Opium also, in the more advanced stages, should the patient complain of restlessness, will be beneficial. Camphor also, in the low state of the disease, has been useful. In the early stage, wine is improper.*

" But, although the use of wine and other stimulants, in the early stage of hospital gangrene, while there is great vascular action as well as much local inflammation, cannot be too highly reprobated, yet there are states of this disease in which it will be found not only not injurious, but very beneficial: such, for example, as the advanced stages of hospital gangrene, which occur in poor old infirm people, or where the patients have lingered long in hospitals, and their health has been broken by previous disease, or where the fever is evidently from the first of a typhoid character." (P. 40.)

*Local treatment.*—Topical applications may be comprised under three heads—sedatives, escharotics, and stimulants.

" When the inflammation continues violent, cold applications, such as water alone, rendered colder artificially, and solutions of sugar of lead, are what I should prefer. Whatever objections there might be to the cold affusion as a general remedy, nothing appears better calculated to subdue inflammation in the local affection, and consequently to allay pain, than the continued application of cold: besides, in very warm weather, it is much more agreeable to the feelings of the patient than any thing hot. To obtain all the advantage from the sedative effect of cold, cloths dipped in the liquid should be applied to the part, and kept constantly moist. I have already mentioned an instance of the good effect of cold in the prevention of hospital gangrene, and I have no doubt that, if applied steadily in cases to which it is adapted, it will be found a most valuable remedy.

" Poultices of all kinds,—the common emollient, as also the fermenting, carrot, turnip, and charcoal,—being always applied warm, have appeared to me in general to aggravate the pain; nor can we be surprised at it, when we consider what a powerful stimulant caloric is. Poultices, though applied cold, soon acquire the temperature of the body: they are, therefore, not the best applications in this complaint, and are objectionable also on account of their weight.

" When the inflammation abates, the sloughs separate, healthy pus is secreted, and florid granulations spring up. When this is the case, the wound should be dressed simply with dry lint, over

* Thomson's Lectures on Inflammation, p. 495.

which a pledget of emollient ointment ought to be applied, and the whole supported by a good compress and roller. Should the sloughs continue to adhere after the inflammation has abated, some stimulating application, such as a mixture of resinous ointment and oil of turpentine, known by the name of warm dressing, may be made to the wound; or an ointment composed of unguent. resinosum and oxyd. hydrarg. rubrum, in the proportion of ʒj. of the latter to ℥j. of the former. On such occasions I have also found the diluted nitric or muriatic acids, or the citric and acetic acids, good applications; in the same cases, a solution of argentum nitratum will be found very useful. It is in this state of the inflammatory gangrene, that warm fomentations and poultices may occasionally be employed with advantage, and that the stronger escharotics, such as the concentrated mineral acids, caustic alkalies, arsenical solution, or the actual cautery, may be used most successfully; but it is not improbable that escharotics, applied at a very early period, when the morbid action is just commencing, may sometimes, particularly in old wounds, arrest at once the progress of the disease. A more generous diet may now be allowed, and even a small quantity of wine. But, although the patient has arrived at the stage of convalescence, and may be considered as safe, I can affirm, from extensive experience on this point, that if he be guilty of any excess, more especially in drinking, of using exercise, of not attending to the proper dressing of the wound, or neglecting the state of the digestive organs, he is almost certain of suffering a relapse, when the same train of symptoms will be renewed, and the danger of the patient will be infinitely greater, and exactly in proportion to the state of debility to which he is reduced." (P. 41.)

The utmost attention must be paid to cleanliness of the wound and proper bandaging. Should phagedæna supervene, which may be known by the appearance of a small dark spot or ulceration, escharotics should be immediately applied. The argentum nitratum and oxyd. hydrarg. rubr. have been chiefly employed by Dr. B. The undiluted sulphuric, nitric, and muriatic acids have also been used with the same intention. Mr. Blackadder is of opinion that hospital gangrene, under any form, may be speedily and certainly cured by the external application of the arsenical solution. The actual cautery has been strongly recommended by the French writers.

The paper terminates by three tabular returns, which give an idea of the extent to which gangrene prevailed in the Peninsula, and of the comparative efficacy of the different modes of treatment.

A paper, by Dr. Ballingall, gives a brief description of a remarkable case of *Crural Hernia.*

*Observations on the Natural or Spontaneous Cure of Syphilis,* by Dr. Wilson, of Hull, is the next communication. The object of the writer is to offer some comments upon the practice of the army surgeons, as the inquiries they have so meritoriously instituted may not convince every mind of the propriety of their practice. Some interesting remarks are made upon the causes most likely to impede the adoption of their treatment; and facts and inferences are stated which seem to confirm the principles on which it is founded. Dr. W. observes, that—

"Whether the use of mercury may be safely superseded altogether in this disease, will require a large accumulation of experience to determine: in the mean time, where the antiphlogistic treatment can be safely adopted, and effectually followed, there can be no doubt that it will be equally successful in private practice; but, as the same rigid observation of rules cannot always be expected, it is probable that the general results will be less favorable than in hospitals, or in military and naval practice." (P. 71.)

*A Case of Polypus, of great size, which was removed from the Root of the Tongue by Ligature,* is next related by Dr. Hue.

Some interesting observations are made in a paper, by Dr. R. E. Grant, *on the Viscera of the common Swordfish.*

Dr. Gibson gives a brief account of the *Epidemic Erysipelas which appeared in Montrose and the neighbourhood in* 1822. This disease continued to prevail epidemically for about four years. It was much more severe in all the symptoms, and more fatal, than the sporadic erysipelas. The mortality was about fifteen per cent. The disease was not so much confined to the head or face as common erysipelas; it frequently occurred in other parts of the surface of the body. The internal fauces were sometimes attacked, and, if it spread to the trachea, it generally proved fatal. In illustration of the nature and progress of this disease, some cases are detailed.

*Case of a Congenital Disease, or Malformation of the Thigh-bone, illustrating the Pathology of Interstitial Absorption of the Cervix Femoris.* By Robert Knox, M.D. &c. &c. The subject of this case was two years old, and died of pulmonic disease. It was suspected that there existed a tendency to scrofula in the family. A striking difference existed in the right lower extremity, when compared with the left. The limb was about three-fourths of an inch shorter than the other, and the toes were slightly turned out. The hip-joint was sound. The acetabulum was not at all affected. The head of the femur was in a natural state, but the neck of the bone might be said to have disappeared. Such cases are rare.

"The continental pathologists, and more particularly Mr. Beclard, seem to regard these alterations, which attack not only the trochanters and neck, but also sometimes the head of the bone (as may be seen in all museums), as arising from the continual pressure and weight of the body. The above case offers an insurmountable objection to this theory; and, besides, I have in my possession the vertebræ of a young person which have undergone this alteration, and in whom all the other bones of the body were perfectly sound." (P. 104.)

*On the sudden Spontaneous Obstruction of the Canals of the larger Arteries of the Body; with some Observations on the Process employed by Nature to prevent or arrest Hemorrhage from lacerated Arteries.* By J. W. Turner, Professor of Surgery, &c.—It has sometimes been observed that the pulse has suddenly and permanently disappeared in one part of the body, while it has continued distinct in the other parts. In some cases of this kind, it has been found, on post-mortem examination, that an obliteration had taken place of a portion of the tube of the artery, in which the pulse could not be felt during life. No full account of this morbid affection has yet been published, and we are indebted to Mr. Turner for the relation of several interesting cases of it, which our limits will not permit us to abstract. Several of them have, indeed, been already published in different works.

From the number of cases he has been able to collect, Mr. Turner is disposed to believe that the sudden spontaneous obstruction of the arteries is not of unfrequent occurrence. The communication is interesting, not only from the mass of evidence collected upon the subject, but from the judicious practical observations which the writer offers. Two plates are given, illustrating the obliterated condition of different arteries.

We are obliged to pass over several papers, which are not, however, without interest.

This volume concludes with some *Additional Cases and Observations, illustrating the Origin of Tubercles,* by Dr. Alison, &c. &c.—It would appear, from many facts which have occurred to Dr. A., both from reading and from personal observation, "that in certain constitutions, inflammation, acute or chronic, but most generally chronic, does frequently and directly lead to the deposition of tubercles. As far as the most minute anatomical investigations can inform us, tubercles are very seldom found in the bodies of children who are stillborn or die very shortly after birth. On this point, Dr. ALISON trusts chiefly to the statements of Dr. DENIS. In by far the greater number of the very numerous cases in which tubercles are found in bodies of young children, the diseased actions by which they were formed had originated after birth, and seldom sooner than some months after birth; parents transmitting to their offspring only the tendency to this kind of diseased action, and very seldom the actual disease. Several cases are detailed for the purpose of confirming the opinion that tubercular depositions are frequently consequential upon inflammatory diseases.

---

*Hints to young Medical Officers of the Army on the Examination of Recruits, and respecting the feigned Disabilities of Soldiers; with Official Documents, and the Regulations for the Inspection of Conscripts for the French and Prussian Armies.* By HENRY MARSHALL, Surgeon to the Forces.—8vo. pp. 224. Burgess and Hill, London, 1828.

THERE are two very difficult duties which the army surgeon is called upon to execute. The first is to prevent men, who offer themselves as recruits, from entering the service with some concealed moral or physical infirmity, which unfits them for the life of a soldier; and the second is to guard against the various, well-conceived, and long-sustained stratagems, by which those who have had enough of a life of glory endeavour to escape from it. We have ourselves frequently felt the perplexing difficulties which attach to either of these services, and should have been very grateful for such numerous and judicious hints as those which Mr. Marshall has given upon this subject.

The design of the author is to explain to young medical officers the nature of the duty of examining recruits, and to illustrate the rules and usages of the service upon that point. The remarks on the feigned disabilities of soldiers are intended to convey some information upon a subject, a knowledge of which can only be thoroughly acquired by a long acquaintance with military hospitals, and a habit of intimately observing soldiers during health, and of treating them under disease.

The heavy responsibility that is attached to the execution of such a duty must be evident. On the one hand, punishment may be inflicted where real disease exists, from the belief that the soldier is a "skulker;" and, on the other, we may suffer ourselves to be duped by artful impostors, to the obvious injury of the service. It is not only the loss of one man that is to be guarded against. The successful attempt is known throughout the regiment, and others, who are equally anxious to escape from military discipline, or to obtain pensions to which they have no claim, are encouraged to repeat it. If it is known that the surgeon has been once deceived, the fear of detection is naturally diminished.

It cannot be necessary that we should dwell at any length upon the necessity of the utmost caution in doubtful cases. By time, and an accurate scrutiny, we may frequently arrive at a just decision, where a hasty opinion would be dangerous; and common humanity demands that every precaution should be used, and the utmost degree of medical skill exerted, to avoid so cruel an error as that of fixing a stigma upon the character of an innocent and suffering soldier, or perhaps even of adding the tortures of the lash to the pains of disease. It cannot be expected that the young army surgeon, however attentively he may have studied his profession, can perform so difficult, and to him so novel a task, with the tact and adroitness of the veteran practitioner, who is well acquainted with all the tricks and stratagems that are frequently so ingeniously conceived, and so ably executed. To him, therefore, caution will be doubly necessary, and the assistance of Mr. Marshall's "Hints" doubly valuable.

We must pass over the "Official Documents," which are placed at the beginning of the work. We cannot, however, but observe, that many of the Horse-Guard "Circulars" were so severely worded as necessarily to lead to almost an indiscriminate rejection of recruits. That such was the effect they produced, may be inferred from the more moderate and qualifying tone which marks those which were subsequently issued.

In two preliminary sections, we find some interesting remarks upon the regulations for the admission of recruits into the French and Prussian services. In the former, conscripts convicted of feigning disabilities were sentenced to hard labour for five years. To secure the fidelity of the executive officers, a host of informers was employed. In Napoleon's time, the price for a substitute was at one period fourteen thousand francs (560*l.*)

The leading qualities required in recruits are comprised under four heads:* viz. height; a certain period of life; health; activity, or the full power of using the several members of the body. The two former belong to the province of military officers: the latter are generally left to the determination of the medical branch of the service. The infirmities or defects that disqualify recruits for service are divided into three classes: obvious defects, chiefly external; defects not obvious, chiefly internal; feigned defects.

*Obvious defects.*—Defects of this class are frequently dissimulated by recruits, who are often instructed in this species of fraud by men belonging to recruiting parties and old soldiers. The military, as well as medical officers, are sometimes imposed upon. Recruits have added to their height by glueing pieces of buff to the naked soles of the feet. Again, the height is reduced, if necessary, by flexing the head forwards a little, pushing out the abdomen, and slightly bending the knees. To dim the brightness of grey hairs, blacking has been applied.

"In the examination of recruits, the following routine will be found to be both expeditious and safe: the names, trades, &c. of the recruits for the day having been inscribed in the register, let them 'fall in,' and be inspected in their clothes. During this inspection we frequently succeed in detecting deserters, and men who have been in the army, and discharged in consequence of disease or disability.

"Let them next be examined singly undressed. Upon entering the inspection room, each recruit is to walk a few times pretty smartly across the apartment, for the purpose of ascertaining that he has the perfect use of his inferior extremities. This is a very essential part of the business of inspection. Notwithstanding a rigorous observance of it, however, I have known a medical officer called upon to explain why he approved of a recruit, who, after joining the corps to which he belonged, did not perform the 'goose step' to the entire satisfaction of his commanding officer. He is then to be halted, set up in the position of a soldier under arms, with the knees about an inch apart, and examined from head to foot. The inspection may be conducted with reference to the following qualities, or conditions of the body:

"Colour. Muscular capability. General health.

"The condition of the external surface, comprehending chronic eruptions, marks of punishment, ulcers, cicatrices, &c.

"The configuration of the thorax, spine, and pelvis.

"The condition of the superior extremities, comprehending symmetry, fractures, contractions, mutilations, &c.

* The specific rules for the guidance of the surgeon were issued in June 1824, from the army medical department. They are given at page 13 of Mr. Marshall's work.

"The condition of the inferior extremities, including symmetry, &c. as also varicose veins, nodes, flatness of the soles of the feet, distorted and supernumerary toes.

"Should no material defect be perceived during this survey, the examination should go on. The recruit is then to perform, in imitation of the hospital sergeant, the following manual evolutions: To stretch out the arms at right angles with the trunk of the body, then touch the shoulders with the fingers, next place the backs of the hands together above the head; in this position let him cough, while at the same time the examiner's hand is applied to the rings of the external oblique muscles. Examine the spermatic chords and testes; then pass the hand over the bones of the legs. The recruit will next stand upon one foot, and move the ankle-joint of each extremity alternately. And when any doubt is entertained respecting the efficiency of the ankle-joint, or any part of an inferior extremity, he should be made to test his strength in that respect by hopping upon the suspected limb for a short period; and the size and aspect of the corresponding joint or part of the opposite limb should also be accurately compared. Let him then extend the superior extremities forward, for the purpose of having his arms and hands examined: he is in this position to perform flexion and extension of the fingers, and to rotate the forearms. The head is next to be examined, including the ears, eyes, nose, mouth; then ascertain that he possesses the function of hearing, and the power of distinct utterance; next inquire whether he has passed through the small-pox or been vaccinated. The examination of a recruit in this manner will require about five or six minutes; and, if carefully performed, very few disqualified men will be admitted into the service." (P. 62.)

No recruit should be examined while he is intoxicated. "If it be discovered that a recruit had formerly been in the army, his case should not be determined until he produce his 'discharge,' or 'instructions,' by which the cause of his leaving the service may be ascertained. Many men, without apparent disease, or any well-marked physical defect, will make but indifferent or bad soldiers.

"There is a very objectionable description of recruits often met with in large cities, namely, young men whose health has suffered from debauchery of various kinds. Their peculiar appearance is commonly well marked: complexion wan and colourless, doughy sodden look, tremulous lips and hands, clean teeth, breath, and smell peculiar to spirit-drinkers; often fulness of the belly and tendency to fatness; their manners and language of the better kind. This class is usually composed of footmen out of place, clerks, shopmen, broken tradesmen, profligate irreclaimable sons of gentlemen, &c. &c. I know of no species of recruits more unfit for the service; they are seldom out of the guard-room or the hospital." (P. 69.)

Marks of punishment, whether military or *civil,* are, in obedience to the rules, sufficient cause for absolute rejection. In the examination, plasters, however small, should be removed from the skin. They are sometimes employed to cover the D which has been made, by the decision of a court martial, for desertion.

*Cicatrices.*—Scars on the neck, the probable consequence of strumous ulceration, are commonly deemed a disqualifying defect. Whether they should invariably cause a recruit to be rejected when he has attained the age of manhood, may admit of doubt.

If the cavity of the thorax is diminished by curvature of the spine, the incapacity of a recruit is evident. Deformity, or any disabling circumstance attending the feet, is a serious imperfection in a soldier. He is soon fatigued, is unable to endure a long march; his feet are liable to swell, inflame, and excoriate. A disposition to hernia, from preternatural enlargement of the ring, is not unfrequent, and forms good ground for rejection. Hydrocele and sarcocele are decidedly disqualifying infirmities. The state of the eyes should be carefully attended to.

Without considerable care, we may be deceived as to the "mental faculties" of recruits. Absolute wisdom, we apprehend, would not be required; but a mere natural, though he might stand coolly enough to be shot at, could never make a soldier, in the true acceptation of the term.

To determine the existence of internal disease, is frequently not an easy task. Many infirmities possess no external mark of their existence; neither are they evident from manifest symptoms. Even after the most rigorous inspection, therefore, unfit recruits will occasionally obtain admission into the ranks.

*Feigned defects.*—The simulation of infirmities is much practised by recruits, as well as by old soldiers. The one wishes to escape admission, and the other to procure a discharge, particularly if he has any chance of sweetening it with a pension.

"Some excite ulcers; others affect stammering, deformity, pain in various parts of the body, deafness, blindness, epilepsy, contractions of the fingers, lameness, &c. Some of these simulators display considerable art in carrying fraudulent plans into execution, and arrange their assumed defects so as to have them in tolerably good keeping." (P. 89.)

Let it never be forgotten that "many recruits, who, from disgust with the service during the period of hard drill, evince a disposition to simulate ailments, or to aggravate trifling defects, become, by mild and humane treatment, excellent soldiers. From the experience which we have had, we are inclined to lament that the initiation of recruits is so completely left to non-commissioned officers, who not unfrequently estimate their importance by the degree of severity they exercise. We freely admit there are many and honorable exceptions to this remark: it will apply, however, but too often.

"Too much care cannot be taken, both by military and medical officers, to make young soldiers fond of their profession. For this purpose they ought to be treated with a due degree of respect, their condition should be rendered as comfortable as strict discipline and circumstances will permit; they ought never to be tormented with useless innovations, or exposed to unnecessary fatigue; every engagement or promise made to them ought to be rigorously observed. Correct discipline should, if possible, be preserved without the adoption of measures that may be denominated severe, or that have a tendency to humiliate or degrade a man in his own opinion, or that of his comrades. While breaches of discipline are punished, good conduct ought never to pass unnoticed. Implicit confidence should rarely be placed in non-commissioned officers with regard to their conduct to the men. Young soldiers are commonly unwilling to prefer complaints, but when they do they deserve a patient hearing. A commissioned officer should himself ascertain that strict justice is given to a recruit in every thing connected with his barrack accommodation, food, and pay. (P. 90.)

The conduct and general demeanour of soldiers are much influenced by the nature of the discipline under which they are controlled. Hence there is a much greater proportion of malingerers in some regiments than others. In considering the subject of counterfeit diseases, or disabilities, two objects are to be considered:

"1. What are the means most likely to be successful in discovering whether an alleged disease be real or feigned?

"2. When a malingerer has been detected, or, in other words, when it is, after due consideration, presumed that a disease is feigned, what are the most probable means for inducing him to return to his duty, or for convicting him? (P. 92.)

In a great majority of instances, an impostor cannot long conceal his deception from a careful observer. Dr. Beck's opinion on this subject is, however, much too strongly stated. He says, "Nothing can be more disgraceful than that a surgeon, one who is supposed to know the nature and symptoms of disease, should be deceived by an individual who feigns his maladies."* To this Mr. Marshall very properly replies, that

* Elements of Medical Jurisprudence, p. 28, English edition.

every medical officer, at all conversant in the wiles of old soldiers, will readily admit that he has sometimes been deceived. There is no circumstance which so commonly distinguishes truth from fraud, as the report a malingerer makes in his complaints. Although he may have gleaned a little information from medical works, in order that he may be enabled to play his part with dexterity, he will usually fall into some inconsistencies which will lead to his detection. We can rarely derive information from the hospital attendants and orderlies: they would incur the hatred of their companions if they were known to communicate privately with the medical officers respecting the conduct of patients. Mr. Marshall suggests as an improvement, that it would be well "to have a ward or two in all general hospitals, on the plan of a *panopticon,*" where a confidential person might observe what was going on, unseen by the patients.

Various infirmities are simulated by soldiers. Intermittent fever; continued fever. To quicken the pulse, tobacco is swallowed, or introduced into the anus; flour and chalk are employed to whiten the tongue. If the bilious tinge of a coated tongue is required, a little gingerbread is chewed. Mr. Marshall has reason to think that ligatures are sometimes applied round the thigh to aggravate, if not to excite, varicose veins of the legs. Various irritants are applied to the eye, to produce inflammation. "To excite disease of the palpebræ, the hairs of the ciliæ are extracted, and caustic applied to the place where they have been withdrawn." In France, above 200 conscripts succeeded in being declared amaurotic by the external application of belladonna to the eye. The same trick is sometimes practised here. The author has known "dilated pupils and blindness temporarily produced by a small portion of the leaf of the *datura metel,* which was mixed with the man's food." Chronic disease of the liver is frequently pretended. It is often extremely difficult to detect the simulation of rheumatism. The non-existence of uneasiness cannot be proved, and all must admit that a considerable degree of pain may be present without a well-marked change in the external appearance. Spitting and vomiting of blood, by various manœuvres, are frequently assumed. A private belonging to the tenth regiment displayed considerable fortitude:

"This man pretended that he had lost the power of his inferior extremities, and for a period of about two years endured all that medical skill and suspicion of his testimony could suggest, with the view of enabling or forcing him to return to his duty. Before recommending him to be invalided, his medical attendant submitted him to the following trial: he was confined in a small room, and a shelf well stored with provisions suspended over his head, which he could easily reach by merely standing upon his legs, but not otherwise. At the end of forty-eight hours, the food remaining untouched, it was not considered advisable to prolong the experiment. He was then included in the list of invalids, and put on board a transport bound for England. While in the harbour, an alarm was given, about midnight, that the ship was on fire; every one hurried into a boat alongside: after reaching the quay, the passengers were mustered, and it was found that the paralytic invalid had not only succeeded in saving himself, but also his trunk and clothes. He was remanded to the ranks. (P. 124.)

Again—

"A soldier asserted that he had nearly lost all power over the inferior extremities, in consequence, as he stated, of a hurt received on the loins. Active means were employed; and, as he was from the commencement suspected of being an impostor, the measures were long continued. The patience of the medical officer who attended him became exhausted, and he was eventually recommended to be discharged. The day he was to receive his discharge, he crawled on crutches to the office where it was to be given him. Having obtained the document, he begged one of the officers of the establishment to read it to him, which he did twice. After satisfying himself that the discharge was properly made out, he first deliberately threw away one crutch, then another, and darted forward, overturning two men who happened to be before him, and finally disappeared, springing over a car, with a water-cask on it, which stood in his way.—During the late war, a man belonging to the Cavan militia was, in consequence of assumed weakness of the inferior extremities, kept in his regimental and the general hospital of this city for two or three years, and almost the whole of this period he never moved without crutches. He was at last discharged. The day after he received his balance of pay, he had himself driven in a car to the Phœnix Park, where the Cavan militia was at exercise. Upon approaching the corps, he laid aside his crutches, and advanced in front of the line; he then bounded like a deer for some time before the regiment, and, after slapping his breech, scampered off as fast as he could. The object of some impostors appears to be incomplete, until they make it known to all their comrades that they have obtained their discharge entirely by a deliberate system of deception." (P. 126.)

Palpitation is occasionally produced by the application of tight ligatures to the neck and upper part of the arms. Irregularity of the action of the heart was in many cases produced at Haslar, by taking the powder of *veratrum album*.

As a proof of the necessity for the utmost caution in doubtful cases of mental disorder, we cite the following lamentable instance of mistaken opinion:

"Joseph Godfrey belonged to the 83d regiment, and served with that corps at the Cape of Good Hope eleven years. During this period he exhibited symptoms of derangement five different times, on *each* of which occasions he was tried by a court-martial for pretending madness in the hope of getting his discharge, and sentenced to be flogged, which sentence was *successively* carried into effect. Maniacal paroxysms continued to recur after he was discharged, and during one of the accessions he committed suicide by drinking a quantity of sulphuric acid." (P. 140.)

Incredible as it may appear, there is good authority for the fact, that, among the French conscripts, ascites was excited by injecting water into the cavity of the abdomen.

Enough has been stated to show how much army surgeons have to guard against, and to prove that, however well qualified for civil employment a practitioner may be, it will be necessary for him, if he enter into the service, to make himself thoroughly acquainted with the duties which are peculiar to it. For valuable suggestions upon many of the most important and perplexing ones, we cannot refer to better authority than the very interesting and instructive volume we have just noticed.

---

*On Difficult Cases of Parturition, and the Use of Ergot of Rye.* By W. Michell, Member of the Royal College of Surgeons.—8vo. pp. 128. T. and G. Underwood, London, 1828.

The author of the present volume does not lead us to expect a complete treatise on the difficult cases of parturition, and still less a system of instruction on the science of midwifery. He gives us merely a few practical hints on the lingering and laborious cases which every accoucheur must occasionally meet with, and in the treatment of which but little assistance is to be derived from professed treatises on the subject of midwifery in general. The materials from which this publication has been written have been collected during many years in the course of the author's practice. Notes have been regularly made of all singular and difficult cases at the time, and of the practice pursued on the occasion; and from the accumulation of these manuscripts the present work has been gradually formed.

Particular attention has been paid to the effects of the ergot of rye, and the author trusts he has *proved* the virtues of this remedy, concerning which much discrepancy of opinion exists. In every obstetrical work, we look as a matter of course for a dash of indignant feeling against the notorious few who have endeavoured to decry the utility of this branch of science, and to degrade the respectability of its professors.

Their efforts, however, have recoiled upon themselves, and the effusions of their spleen might safely be passed over as unworthy of any formal notice. Upon this subject but one observation appears to be necessary. The very men who assure the public that uneducated females are quite as capable as the well-instructed male practitioner of performing the various duties of the obstetric art, give a flat contradiction to their own assertions, by securing the assistance of the latter in their own families. Dr. KINGLAKE, of Taunton, it appears from Mr. MICHELL's preface, "accuses the medical practitioners of having cajoled the women into the indelicate system of retaining male attendants on such occasions." The delicate Doctor is recommended to make inquiries in his own neighbourhood, to ascertain what sort of cajolery has been practised; and, if he fails in obtaining this knowledge at home, he may consider what effect the following statement from Mr. Michell's neighbourhood would produce in cajoling the females into the modern system: "Of twenty-one deaths that have occurred in this neighbourhood within the last twelve years, *nineteen* have been women attended by females only!

Mr. Michell complains, unjustly we think, that the subject of puerperal convulsions "has been most sadly neglected by most writers." Has he referred to DENMAN, BURNS, MERRIMAN, DEWEES, &c. &c.? From this assumed neglect of this important subject, he is led to pursue it somewhat further, in the hope that it may be found acceptable to the junior members of the profession. After such an introduction, an elaborate essay upon this disease might be expected. But seven pages, however, are devoted to it, and one case is detailed; and, as we discover nothing important in the preliminary observations, and by no means approve of the treatment of the case, we shall pass over this "further" investigation of the subject of puerperal convulsions with one short extract: After delivery, "the convulsions still continued, with longer intervals. I gave her ℈j. of musk, to be doubled every paroxysm until they ceased. Three doses I have invariably found to overcome the complaint."

The contents of the second chapter are not of sufficient importance to arrest our attention.

Upon the subject of Lingering Labour, Mr. Michell observes, that it will frequently exhaust a woman's strength when the presentation is right, notwithstanding all our attention. That such cases sometimes occur, we do not deny; but our own experience would lead us to doubt their *frequency* under proper management.

A long confinement of the head in the pelvis is equally dangerous to the mother and to the child: by its pressure, it stops the circulation, bruises the soft parts, and often brings on sloughing and gangrene.

"When dangers of this sort occur, I would not advise the adoption of the forceps, as I verily believe that more lives have been lost than saved by the use of them. Because delivery may be expedited by them, they are often had recourse to without the slightest occasion; and, from the cases I shall hereafter introduce, it will appear that, whilst we have medicines which will expel the child naturally, there can be no good reason for the use of an instrument, which often occasions sloughing and death. When there is scarcely sufficient room without the forceps, it is evident that such an addition to the diameter of the child's head must greatly increase the difficulty, and in the same proportion augment the danger." (P. 18.)

We would by no means advocate the hasty application of instruments in the practice of midwifery, but we cannot consent to this condemnation of the use of the forceps in lingering cases. That they are sometimes unnecessarily applied, and sometimes inexpertly, is doubtless true; but this complaint might be urged against every remedy and every instrument we possess. In skilful hands, and in pressing cases, the forceps is both a safe and an effectual instrument. With respect to any medicine capable of inducing the natural expulsion of the child, much additional testimony must yet be presented to us before we should venture to depend so confidently upon its agency as Mr. Michell is inclined to do.

Upon the subject of the Cæsarian operation, the author argues that no valid reasons can be alleged for a preference to this operation to the opening of the child's head.

"I am at a loss for the least excuse for the practitioner, who would sacrifice the life of the mother that the child may be saved. To say nothing of the great danger of sacrificing both by so rash a choice; for I conceive the most resolute of our profession would hesitate long before he made up his mind to so cruel an expedient, and this delay would generally insure the death of the child. But, supposing the infant could be saved, on what plea can we put the life of the mother in comparison with that of the child? If, by a contrary practice, we save one woman out of five, even to the destruction of five children, I conceive the advantage is still on this side. If the increase of mankind be the consideration, the woman saved may afterwards have five children, and these have children as early (within a few years) as the infant had it been saved. Again, the woman may have a family, and thus a valuable life would be placed in the greatest jeopardy for the sake of saving an infant, which, in the lower ranks of life, if deprived of its mother, generally becomes a burden to every one concerned with it, and often a pest to society, from its want of maternal protection in his youth. I may then be allowed, I should hope, to plead the cause of the unfortunate mother, and to urge, as I most earnestly do, that every chance of life should, in all cases, be given to the woman.

"I would, therefore, in every instance, recommend the opening of the child's head, in preference to the Cæsarean operation; and we shall then, at least, not have to mourn over orphans deprived of a parent's care, by any calamity which our art might have prevented. Let the medical practitioner then first open the child's head; endeavour to produce expulsive pains, by the means I shall hereafter point out; and, should these not be attended with success, let the head be cut in pieces, which may be easily effected: it may, indeed, without difficulty be pulled in pieces, so as to pass some of the most narrow pelves. And let us hear no more of the Cæsarean operation, which I consider a disgrace on the profession of the accoucheur, as scarcely an instance of recovery from it has occurred in modern times." (P. 22.)

Mr. Michell informs us, that if he did not object to the Cæsarean section in every case, he would point out a more safe method of performing it before the head descended into the pelvis, and even after, than the plan now recommended. "In all the recorded cases which we can rely on as true hysterotomy, or cutting through the uterus, the deaths have been fifty to one recovery." Mr. Michell would therefore pronounce it to be justifiable only in the instance of the woman's death. In that case it may save the child, if performed with sufficient expedition. We freely confess we have no practical knowledge of this dreadful operation, but we can conceive cases of extreme deformity where it would be impossible to deliver the woman by any means without having recourse to it. We lament, therefore, that the author has not given us the opportunity of judging of the superiority of the mode of performing it to which he alludes. Dr. Dewees has taken a very judicious view of this subject in his System of Midwifery.

We consider it particularly fortunate that Mr. Michell is in possession of a remedy for exciting the action of the uterus, upon which he places so much reliance, namely, the ergot of rye; for it appears that, before he trusted to the effects of this medicine, he was in the habit of gradually introducing his hand, when he "found the os uteri thick, rigid, and unyielding, or deep, thin, and flaccid," until he had fully dilated the parts. Cases may certainly occur in which this sort of manual interference may be justifiable for the purpose of hastening delivery; but to lay it down as a general mode of practice in such states of the soft parts, we hold to be alike dangerous and injudicious. We would rather, with Dr. CONQUEST, do nothing, than run the risk of doing irreparable injury. Upon this subject, indeed, there appears to be a want of agreement in Mr. Michell's doctrines and practice; for in the next chapter we find no mention of the introduction of the hand in a case of lingering labour from rigidity of the soft parts. Has Mr. Michell no confidence in bleeding, mild laxatives, emollient injections, and *patience* on the part of the practitioner, where the os uteri is "thick, rigid, and unyielding?"

We are again compelled to dissent, and strongly too, from the opinion that diluted spirits "were the best means we were possessed of to accelerate lingering labours, before we were acquainted with the good effects of the ergot of rye."

To increase and quicken uterine action, and thereby to facilitate delivery, the author has tried various vegetable* and mineral substances. The former were sometimes of limited use; the latter produced no effect whatever. He therefore very properly paid immediate attention to the accounts of the ergot of rye, and subjected its pretensions to the test of his own experience. Of this remedy he gives a very favorable opinion.

"The result has been, that, after its successful application for many years, I am now fully convinced that it is a safe and efficacious medicine in accouchery cases; possessing all the properties which a practitioner could desire. Its effect on the contraction of the uterus, both before the expulsion of the fœtus and after this has been accomplished, is such as to remove all danger attendant on the woman, quickening and facilitating the delivery, and preventing spasm or flooding afterwards." (P. 54.)

We pass over the enthusiasm with which the author proceeds to dwell upon this subject. He is of opinion that, as soon as the ergot of rye is generally known in female practice, "it will supersede the necessity for male practitioners, except in a very few instances." He will not be surprised if, in twenty years, the forceps are known only by name. Is he serious?—Ergot of rye to be administered by a drunke[illegible] woman

In the eighth chapter, a succinct account is given of the production of the ergot from rye and other plants; its general appearance, as stated by different writers, and observed by the author; its medical properties; when first observed, and the difference of opinion with respect to its poisonous qualities. That particular property of ergot which forms the

* What were these vegetable substances?—REV.

subject of this work, its power of exciting the uterus and increasing its action, was first noticed by Dr. Stearns, of New York, in 1807. Until the last three or four years, this medicine has been almost exclusively used by the American physicians. We have so frequently alluded to the subject, that it would be needless, upon the present occasion, to dwell upon the various opinions entertained either of its mode of action or its efficacy. We shall confine ourselves to the result of our author's experience.

In reference to the cautions which have been given for the administration of this remedy, Mr. Michell "hopes that the perusal of this work, and the great number of cases detailed, will satisfy the profession that it will be productive of evil in no cases except those of bad conformation, and wrong presentations which require turning." It appears to act more quickly, and with more certainty, when given in the form of infusion or decoction. Half a drachm is considered the proper dose to commence with. Mr. Michell has given it in wrong presentations, purposely to witness its effects, and has even turned whilst its action on the uterus has been most marked. He therefore does not admit the conclusion, which has been stated by some practitioners, that it is productive of injury to the mother and child where the birth is delayed by mechanical impediment.

"The great value of the ergot consists in its efficacy in cases of non-dilatation of the os uteri: in these its power is most conspicuous, as it will produce its dilatation in a few minutes, which otherwise would require many hours. The repetition of the dose I have not found necessary in more than one case in fifty: to require it at all, there must be a great rigidity of the ligaments, muscle, and, in fact, of all the parts both within and without the pelvis." (P. 71.)

In abortion, Mr. Michell considers the ergot altogether inefficacious.

By many, the use of this medicine has been restricted to cases of natural presentation, where the parts were properly dilated, and the progress merely delayed for want of action. But in such cases we had, says the author, all we required without the ergot; for one and a half ounces of brandy in six ounces of water, *repeated every fifteen minutes,* would be certain in a short time to bring on uterine action. In the lower ranks of society, gin and brandy are generally administered with as much liberality as even Mr. Michell recommends; and yet cases of lingering labour, where the soft parts are fully dilated, are quite as common in this class of patients as amongst those who have too much discretion themselves, or who are at least too much under the guidance of judicious practitioners, to adopt the reprehensible use of spirituous potations, which we here find so unwarrantably advised. Let us not be accused of undue or unnecessary severity; but we cannot refrain from entering our protest against this encouragement of a most injurious practice, which every accoucheur must have frequently found it so difficult to restrain, and of the evil effects of which most are fully sensible. We admit that the administration of brandy may occasionally be required during the progress of labour; but its use must be limited by much judgment and discrimination.

It has been said that we have no power of controlling the effects of the ergot when they are once established. The author denies this assertion. "Opium will be found to stop its effects within a very few minutes." The only distressing symptom Mr. Michell has ever known this remedy to bring on has been vomiting, which he considers almost a certain attendant on the rapid dilatation of the os uteri.

"Another objection is, that when the child is born any length of time after its administration, it is always still-born. This opinion of its effects I consider to be altogether erroneous. The reason why the death of the infant has been attributed to it is, that it is only administered in long, lingering cases, in which it is well known the child is frequently stillborn without the exhibition of ergot." (P. 73.)

Flooding has been said to cause death when ergot is administered. Mr. Michell contends that death from flooding, where the ergot has been given, can never occur. "It is, on the contrary, of the greatest advantage in cases of flooding, and were it only for its virtues in hemorrhage it would be a most invaluable medicine."

The volume concludes by a detailed statement of many cases in which Mr. Michell has given it with complete success, and of *all* those in which he considered it to have failed. He trusts that, from the facts he has stated, "every one will be able to judge for himself of the powers of ergot without attending to the opinions, arguments, or theoretical deductions of any individual whatever. Practice, and practice only, must eventually decide on all subjects of this nature." Now, for our parts, we should be much inclined to weigh well, at least, the opinions and arguments of every individual, in order that we might arrive at a satisfactory conclusion by impartial inferences from the collective evidence which has been accumulated upon this subject.

In most of the cases related by Mr. Michell, the influence of the ergot in accelerating uterine action appears to have been decided. In several instances the os uteri was not at all dilated, and the labour not making any progress at the time of its administration. Some of the cases adduced are not altogether satisfactory as far as regards the agency of the ergot. Some cases of menorrhagia are mentioned, in which no benefit was derived from the use of this remedy. The author states that he has used the ergot in many hundred cases, and that he has never once regretted its use. It may be proper to remark, that he always adds one-third of milk to the infusion, and milk has been said to be antidotal to its violent effects.

In conclusion, Mr. Michell observes that his only motive for undertaking the trouble of this publication is a firm conviction of the utility of the ergot. Upon this subject his testimony is certainly valuable.

---

*Notes on the Epidemic Cholera.* By R. H. Kennedy, M.D. lately Surgeon at Bombay Presidency.—8vo. pp. 277. Calcutta.

Dr. K. informs us that he has been many years a resident in the East Indies, where, since 1818, he has had frequent opportunities of studying the nature of epidemic cholera; that he adopts the opinion that it is capable of being communicated by contagion; and that he believes it to be *concussion of the brain.* We have examined the foundation of his theory with much attention, and we do not find, amongst the arguments by which he attempts to prove his extraordinary notion, any that are original; and the greater number of them are, indeed, those identical ones which Assistant-surgeon Chapman* advanced in 1821, to show that cholera was a disease, probably inflammation, of the cerebellum and medulla oblongata. Dr. Kennedy enumerates many symptoms which strongly support the opinion that cholera may be a primary disease of the nervous system; but he fails in his attempts to demonstrate that the prominent and diagnostic symptoms of acknowledged concussion of the brain are also the most constant and distinguishing ones of cholera. We have ourselves seen instances of both these diseases in India during the last epidemic, and we are surprised that Dr. K. should attach so much importance to the occurrence of the vomiting and collapse in both cholera and concussion, and apparently quite forget that, in cases of concussion, the vomiting is only at the commencement, and is unattended with the peculiar purging so distressing in cholera; that, in

* See Madras Report, 1824.

the latter, the mental faculties are unimpaired and vigorous to the last; while, in the former complaint, they are always more or less injured during the disease, and sometimes continue weak and defective through the remainder of life. Again, debility has been regarded as a very common predisposing cause of cholera, because it is less fatal to Europeans and robust natives than to those whom superstitious rites, intemperate habits, or former disease, have rendered weak. But, as far as we are aware, of these various causes, intemperance alone can be reasonably supposed to aggravate the effects of concussion. The rapid abstraction of blood, the introduction of a bougie, the fumes of tobacco, &c. &c. will all frequently produce long-protracted syncope, with vomiting, &c.; and yet we imagine Dr. K. would not, in these cases, attribute such symptoms to concussion, unless, like Broussais, he admits of but one disease.

Dr. Kennedy does not profess to be the inventor of a new treatment for this disease, as is evident from the following brief quotation, which contains, in his own words, his peculiar doctrines, and the mode of treatment he has adopted:

"I consider concussion of the brain to be the disease, how induced I know not, following the above inexplicable shock sustained by the constitution; and the collapse and spasms to be symptomatic of the disorder of the brain; and, finally, I consider the purging and vomiting to be no part of the disease, but the struggle and efforts of nature to relieve the constitution, and cast off the noxious principle which is destroying it. For the treatment of such a disease, the indication is distinctly apparent to relieve the brain by bleeding, and to induce the sanitary process of vomiting and purging where they do not exist, or to moderate them when violent. Into these brief injunctions may be resolved all that has been written on respectable authority; and the only difference in my theory is, that I would propose a regular systematic procedure, in preference to the uncertainty, hesitation, and undecidedness, which, in spite of every thing that has yet been written, continues to prevail in a case where, of all others, the patient's safety most mainly hinges on the promptitude of treatment."

In justice to the author we must add, that, though we feel quite averse to adopt the peculiarity of his theory, yet we fully appreciate his practical tenets, and consider them very judicious and correct. The "Notes on Cholera" are evidently the production of a man of reading and observation, though the style is too frequently digressive and diffuse: in our opinion, they are highly deserving the attentive perusal of those who design to practise in the East Indies.

